# The GPIb-IX complex on platelets: insight into its novel physiological functions affecting immune surveillance, hepatic thrombopoietin generation, platelet clearance and its relevance for cancer development and metastasis

**DOI:** 10.1186/s40164-022-00273-2

**Published:** 2022-04-02

**Authors:** Gerd Bendas, Martin Schlesinger

**Affiliations:** 1 Department of Pharmacy, Rheinische Friedrich-Wilhelms-University Bonn, An der Immenburg 4, 53121 Bonn, Germany; 2 Federal Institute for Drugs and Medical Devices (BfArM), Bonn, Germany

**Keywords:** GPIb-IX complex, Platelets, Bernard-Soulier syndrome, Thrombin receptor, Mechanosensitive domains, Platelet biogenesis, Platelet clearance, Thrombopoietin secretion, Liver cancer development, Cancer metastasis

## Abstract

The glycoprotein (GP) Ib-IX complex is a platelet receptor that mediates the initial interaction with subendothelial von Willebrand factor (VWF) causing platelet arrest at sites of vascular injury even under conditions of high shear. GPIb-IX dysfunction or deficiency is the reason for the rare but severe Bernard-Soulier syndrome (BSS), a congenital bleeding disorder. Although knowledge on GPIb-IX structure, its basic functions, ligands, and intracellular signaling cascades have been well established, several advances in GPIb-IX biology have been made in the recent years. Thus, two mechanosensitive domains and a trigger sequence in GPIb were characterized and its role as a thrombin receptor was deciphered. Furthermore, it became clear that GPIb-IX is involved in the regulation of platelet production, clearance and thrombopoietin secretion. GPIb is deemed to contribute to liver cancer development and metastasis. This review recapitulates these novel findings highlighting GPIb-IX in its multiple functions as a key for immune regulation, host defense, and liver cancer development.

## Background

Blood platelets are megakaryocyte-derived highly reactive cells that crucially contribute to hemostasis, but also play a pivotal role in thrombosis, inflammation, and cancer metastasis. Upon vascular injury, platelets immediately interact with components of the subendothelial matrix via adhesion receptors to seal the wound. By this interaction, platelets are activated and recruit more platelets to the growing plug. Additionally, activated platelets promote the conversion of prothrombin to thrombin and offer a site for the assembly of coagulation factors resulting in elevated thrombin generation [1–3]. The glycoprotein (GP)Ib-IX complex is the second most abundant adhesion receptor on platelets and solely expressed on megakaryocytes and platelets. Compared to other platelet receptors, GPIb-IX has the unique ability to mediate adhesion and thrombus formation in damaged vessels under high shear conditions like those in small arterioles and sites of arterial stenosis. In addition to this, GPIb-IX is involved in several physiological and pathophysiological processes. This review provides a summary of recent findings concerning the role of GPIb-IX in platelet biogenesis, thrombopoietin production, and platelet clearance. Furthermore, the immunological function of GPIb-IX for instance in leukocyte recruitment, bacterial clearance, neutrophil extracellular trap formation and cancer development are evaluated. Finally, this review describes the concepts of GPIb-IX signal mechanotransduction and GPIb-IX as thrombin receptor.

## Main text

### Structure of GPIb-IX

**Fig. 1:**
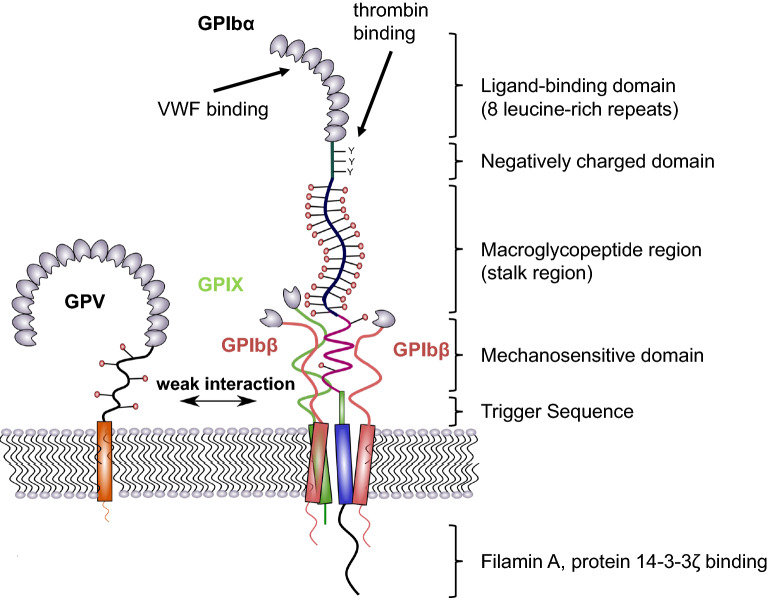
Structure of GPIb-IX. The largest subunit of the complex is GPIbα, which consists of a N-terminal binding domain (made up of 8 leucine-rich repeats), a negatively charged domain, a sialomucin region (macroglycopeptide domain), the mechanosensitive domain (MSD), and proximal to the membrane, the trigger sequence. Intracellularly, GPIbα interacts with protein 14-3-3ζ and filamin A which links the complex to the cytoskeleton. The transmembrane domain provides a close interaction with GPIbβ and GPIX to form a stable parallel four-helical bundle structure. This complex structure is further strengthened by the interplay of GPIbβ and GPIX extracellular domains with the MSD in GPIbα. GPV is weakly associated with GPIb-IX by polar interactions and can be replaced by other receptors

The GPIb-IX complex consists of the three subunits, GPIbα, GPIbβ, and GPIX organized in the platelet membrane in a ratio of 1:2:1. Each subunit belongs to the leucine-rich repeat protein family, containing a short cytoplasmic tail, a transmembrane, and an extracellular glycosylated domain. Both GPIbβ subunits form disulfide bonds with GPIbα and are non-covalently linked to GPIX [4]. GPV, which contains 15 leucine-rich repeats, a transmembrane domain, and a short cytoplasmic tail is weakly associated with the GPIb-IX complex by transmembrane domain interactions with GPIbα. Disruption of this interaction significantly alters the GPV surface expression [5]. GPIbα is the largest subunit of the GPIb-IX complex with a mass of 135 kDa and mediates binding to almost all known ligands. The extracellular N-terminal domain of GPIbα consists of eight leucine-rich repeats that form an elongated shape and are exposed well above the platelet surface forming the ligand binding domain (LBD) (Fig. 1) [6]. Closed to the leucine-rich region, a negatively charged portion containing three sulfated tyrosine residues is primarily important for thrombin binding [7–9]. This domain is connected to a polymorphic mucin-like macroglycopeptide that contains sialylated *O*- and *N*-glycosidically linked carbohydrate chains accounting for 40% carbohydrate per weight of GPIbα [10, 11]. It provides the LBD an exposed position and facilitates ligand binding events. A mechanosensitive domain (MSD) is adjacent to the macroglycopeptide region proximal to the platelet membrane [12]. The MSD can be cleaved by the metalloproteinase ADAM17, which leads to the release of soluble GPIbα fragments (also known as glycocalicin) into the plasma [13]. The recently identified trigger sequence is located between the MSD and the transmembrane domain and is composed of 10 residues [14]. The cytoplasmic tail of GPIbα contains several binding sites for intracellular signaling molecules and links the receptor complex to actin filaments of the cytoskeleton [15, 16]. It was also speculated that platelets contain a large intracellular pool of GPIbα, but this has not been verified so far [17]. Besides VWF and thrombin, GPIbα also binds to P-selectin, the integrin MAC-1 (α_M_β_2_), coagulation factors XI and XII, high molecular weight kininogen, and thrombospondin [18–24].

In contrast to GPIbα, GPIbβ is much smaller containing a single LRR (25 kDa) that interacts intracellularly with calmodulin. GPIbβ is palmitoylated on cysteine residues in the transmembrane region [25, 26].

GPIX is the smallest receptor subunit (17 kDa), which does not associate with any intracellular molecule [27, 28]. GPIX has a cytoplasmic tail of only eight residues, containing a myristoylated one at the junction of the transmembrane and cytoplasmic domain, which potentially localizes the receptor in lipid rafts [26, 28]. Notably, the transmembrane domains of GPIbα, GPIbβ, and GPIX, rather than the cytoplasmic regions are responsible for the GPIb-IX complex expression and assembly and form a stable parallel four-helical bundle structure [29, 30]. The stability is further increased by interactions between the extracellular domains of GPIbβ and GPIX with the MSD of GPIbα [31]. GPV is weakly associated with the complex mainly mediated by polar interactions in the transmembrane domains of GPIbα and GPV and susceptible to non-ionic detergents [5, 28, 32]. Thus, GPIb-IX has the ability to interact with other membrane receptors than GPV.

### GPIbα as thrombin receptor

First observations of the involvement of GPIb in thrombin-mediated platelet aggregation date back to 1976 showing glycocalicin, a soluble fragment of GPIb interfering in platelet aggregation induced by both thrombin or ristocetin [33]. Later, the cleavage of GPV as the only detectable membrane surface protein hydrolyzed by thrombin was revealed [34]. In 1986, a model was proposed in which thrombin binds to GPIb providing an orientation to thrombin for subsequent cleavage of other platelet membrane receptors in proximity to GPIb [35]. Thus, three different binding sites on the platelet surface for thrombin were identified by analysis of thrombin binding isotherms. Protease-activated receptor-1 (PAR-1) was identified as a member of seven-(pass)-transmembrane domain G protein-coupled receptors, which is cleaved by thrombin at the N-terminal end [36, 37]. Upon cleavage by thrombin, a new N-terminus is exposed that acts as a tethered ligand and induces receptor activation and finally a pronounced platelet activation. The identification and characterization of PAR1 shed critical light on the role of GPIb as a relevant thrombin receptor [38].

Notably, also PAR-1 deficient platelets strongly respond to a thrombin treatment, which highlighted the existence of further thrombin receptors besides PAR-1. Finally, three more PARs were identified [39–42], which act in a two-receptor system for platelet activation by thrombin. In murine platelets, PAR-3 is necessary for thrombin responses, supported by a second PAR-4-mediated signaling mechanism [42]. In contrast, human platelets express PAR-1 and PAR-4, and activation of one of these receptors induces platelet activation and aggregation. However, thrombin affinity to PAR-4 is much lower (EC_50_ of about 5 nM) compared to PAR-1 (EC_50_ of about 0.2 nM) [43].

As a consequence of these findings, the implication of GPIb as thrombin sensitive receptor for platelet activation has been regarded controversial for several years. Especially under consideration of findings from Kahn et al. who exhibited that simultaneous inhibition of both PAR-1 and PAR-4 virtually ablated platelet secretion and aggregation [44].

However, there are some reasons in support of GPIbα as a thrombin receptor. First, 20.000–50.000 copies of GPIbα are expressed on platelet membranes which is 10 to 100-fold higher than that of PAR-1 expression [32, 38, 45–47]. Second, the presence of GPIbα obviously facilitates full platelet activation by thrombin. Notably, De Candia and coworkers showed that thrombin binds with high affinity to GPIbα on platelets. This accelerates the hydrolysis rate of PAR-1 suggesting that GPIbα functions as a coreceptor for PAR-1 activation [48]. Furthermore, binding of thrombin to GPIbα protects thrombin from heparin mediated inhibition by antithrombin III [49].

In fact, Adam et al. confirmed that GPIbα is indeed a highly relevant receptor for thrombin-induced platelet activation working in concert with PAR-1. Platelet activation elicited by low thrombin concentrations and PAR-1 cleavage was reduced when GPIbα was blocked. On the contrary, high levels of thrombin in presence of PAR-1 antagonists induced a PAR-4 coupled platelet response that was not susceptible to GPIbα inhibition. Obviously, PAR-1 requires GPIbα as a cofactor for thrombin signal amplification, whereas PAR-4 works independently of GPIbα in the presence of higher thrombin levels [50]. Likewise, in a model based on the immobilization of catalytically inactivated thrombin and independent of PAR activity, GPIbα binding to inactive thrombin evoked platelet adhesion, spreading, dense granule secretion, and integrin ɑ_IIb_ß_III_ controlled platelet-platelet interaction [51]. These results are corroborated by earlier findings detecting platelet activation by catalytically inactive thrombin in the presence of polymerized fibrin in a GPIbα-dependent manner [52]. Moreover, it is well established that other ligands in addition to VWF can induce GPIbα-dependent signaling that culminates in platelet activation [28].

Two different crystal structures of thrombin and the N-terminal domain of GPIbα revealed that one molecule thrombin can bind to two adjacent molecules GPIbα, although the postulated binding modes were different. Thus, it was proposed that thrombin induces an intramembranous clustering of GPIbα which initiates platelet activation. Alternatively, thrombin can crosslink two adjacent platelets via GPIbα binding and promotes aggregate formation [53, 54]. In another study, GPIbα amino acid Tyr^276^ was substituted with Phe^276^ in a transgenic mouse model. The platelets with the Phe^276^ variant were significantly limited in thrombin binding but displayed normal binding to collagen and human VWF. In in vivo Ferric chloride-induced thrombus formation model, mice exhibited a delayed time to occlusion and in a laser injury model thrombi were less stable compared to wild-type mice [55]. To decipher the contribution of PAR1, PAR4, and GPIbα to thrombin-induced platelet activation, Torti et al. desensitized PAR-1 and PAR-4 on the platelet surface and cleaved GPIbα with the cobra venom mocarhagin concomitantly. Surprisingly, thrombin was still able to induce a residual, but evident aggregation, which appeared delayed [56]. Additionally, in PAR1- and PAR4-desensitized, mocarhagin-pretreated platelets, thrombin still induced normal actin polymerization and cytoskeleton assembly. Consequently, the authors postulated an additional, hitherto unidentified thrombin receptor on platelets that elicit platelet activation and depend on the thrombin catalytic activity.

Besides GPIbα, GPV in the GPIb-IX-V complex is a substrate of thrombin, too, and is cleaved in the early phase of thrombin-induced platelet aggregation [34]. Platelets from GPV-deficient mice reacted hyper-responsive to thrombin stimulation and revealed an increased fibrinogen binding and aggregation response compared to wild-type platelets [57, 58]. Thrombosis was inducible in GPV null mice in response to proteolytically inactive thrombin, whereas thrombosis occurred in both genotypes (wild-type and GPV knockout) in response to active thrombin. GPV-deficiency was also associated with a shorter bleeding time. Thus, these data suggest a role for GPV as a negative modulator of platelet activity that upon cleavage unlocks the GPIbα thrombin receptor function.

**Fig. 2:**
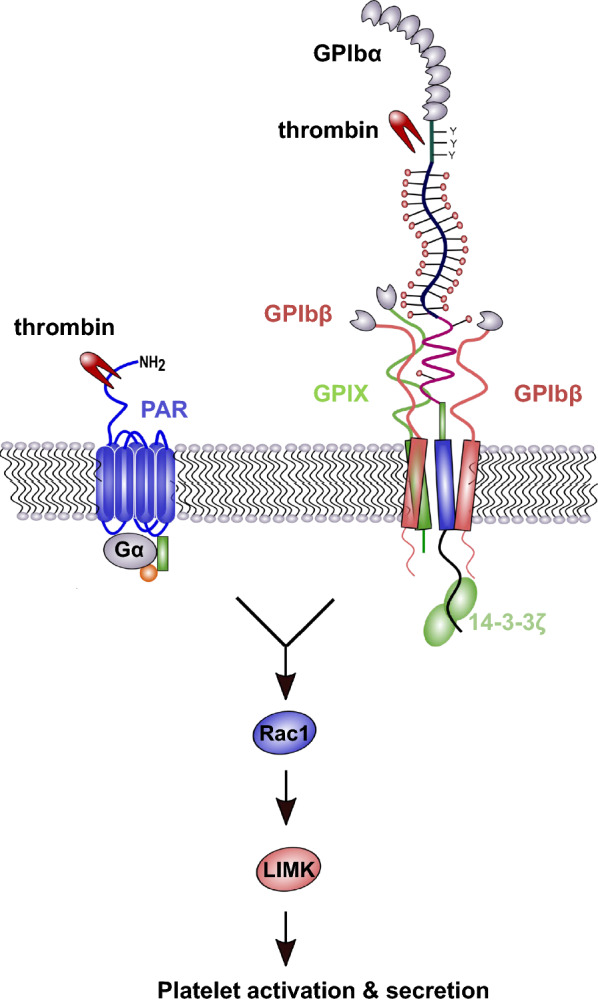
GPIb-IX and protease-activated receptor-1 (PAR-1) signaling cooperation. GPIbα and PAR-1 reveal a mutual dependence for platelet activation by low thrombin concentrations. For full platelet activation, PAR-1 signaling and simultaneously a GPIb-IX–specific 14-3-3–Rac1–LIMK1 signaling pathway activation are required

However, the exact role of GPIbα as a PAR-1 independent thrombin receptor remained a subject of debate for years. On the one hand, GPIbα is considered as a simple, high-affinity dock for thrombin facilitating cleavage and activation of PAR-1, whereas on the other hand GPIbα is regarded as independent thrombin receptor that is even sensitive to catalytically inactive thrombin. Estevez et al. introduced a novel and intriguing concept of cooperativity between GPIbα and PAR-1 that drives platelet activation in response to low-dose thrombin [59]. To study the cooperativity, they generated a CHO cell line merely expressing recombinant human GPIb-IX and PAR1, but not PAR4 or other platelet receptors. They confirmed GPIbα and PAR-1 in a mutual dependency to induce optimal platelet response to low thrombin concentrations. The authors demonstrated that thrombin initiates a GPIbα 14-3-3-Rac1-LIMK1 signaling pathway, and activation of this pathway also requires PAR-1 activity (Fig. 2). Blocking one of the receptors or the intracellular binding between GPIbα and cytoplasmic 14-3-3 reduced the activation of the signaling cascade and calcium mobilization. Catalytically active thrombin was required to induce simultaneous activation of PAR-1 and GPIbα. This novel concept of receptor cooperativity helps to partially explain findings of previous studies and, additionally, highlights GPIbα as a real thrombin receptor rather than a passive dock that joins PAR-1-mediated platelet activation in response to low-dose thrombin.

### Mechanoreceptor domains in GPIbα

GPIb-IX complex has been regarded as the platelet mechanosensor for decades, although the exact mechanism by which the GPIb-IX complex senses shear stress has remained ambiguous [60, 61]. In 2015, two distinctive domains in GPIbα were identified that unfold upon ligand binding and finally transduce force into platelet activation. First, Zhang et al. identified a juxtamembrane mechanosensitive domain in the stalk region of GPIbα between the macroglycopeptide and the transmembrane domain, that unfolds upon von Willebrand factor (VWF) domain A1 binding and pulling [12, 62]. Although this mechanosensitive domain is structured, it is relatively unstable. Mutations abolish a force-induced unfolding of the receptor indicating that no other domain can act as an alternative mechanotransducer.

VWF interaction with the N-terminal domain of GPIb-IX together with a drag force of 5–20 pN induces an unfolding of the mechanosensitive domain and subsequently a conformational change in the adjacent extracellular domains of GPIbβ and GPIX, which initiates a signal through the platelet membrane into the platelet cytosol. The force-induced unfolding of the mechanosensitive domain and consequences for platelet signaling have recently been characterized in detail. In pulling-relaxation experiments the authors showed that refolding of the unfolded mechanosensitive domain occurs also under force and that the glycosylation status of the mechanosensitive domain has an impact on the required unfolding force, e.g., a non-glycosylated mechanosensitive domain needs a significantly weaker force to unfold than the glycosylated one [63, 64]. Furthermore, a longer shear force exposure (up to 5 min) initiates an enhanced P-selectin, and phosphatidylserine exposition on platelet membranes than a 30 s force exposure. These findings support the idea that a distinct tension for a certain period of time is needed to maintain unfolding of the mechanosensitive domain and to activate the GPIb-IX complex.

**Fig. 3:**
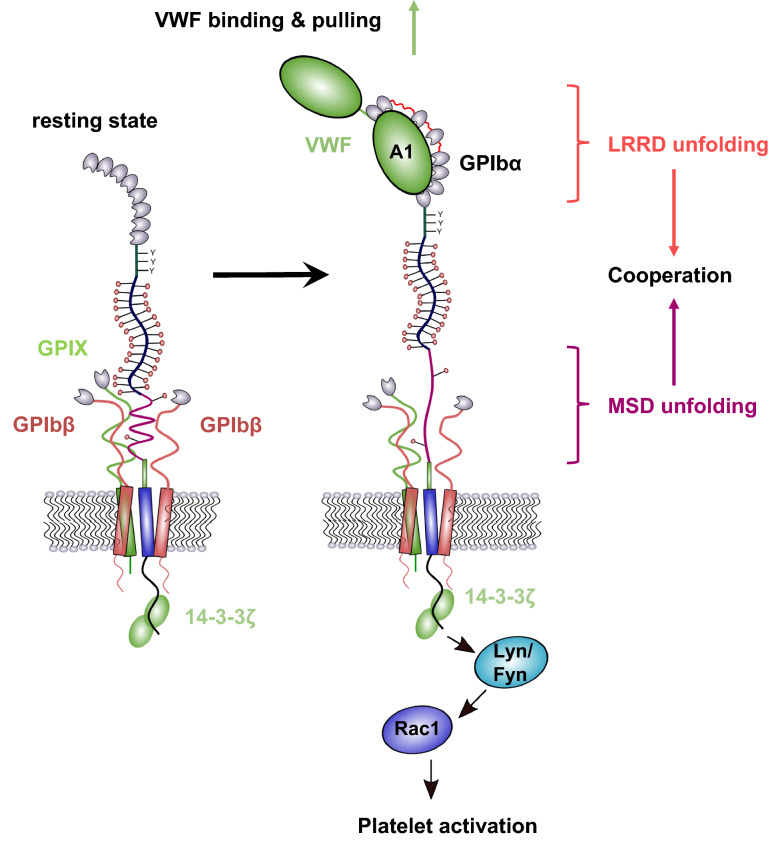
Mechanoreceptor domains in GPIb. GPIbα contains two mechanosensitive domains (MSD), which support each other in signal transduction. The first domain is located in the leucine-rich repeat domain (LRRD) and the second one is between the macroglycopeptide domain and the trigger sequence. Upon VWF binding and pulling, both domains are unfolded and induce a cooperation for intracellular Ca^2+^ release, Rac1, LIMK, and platelet activation

The second mechanosensitive domain in GPIbα has been identified in the leucine-rich repeat domain (LRRD) [65]. The N-terminal domain of GPIbα consists of eight leucine-rich repeats that forms a concave shape. This grabs VWF A1 domain with a N- and C-terminal contact whereas the middle section (LRR 2-4) of the LRRD is surprisingly not involved in binding [6, 66]. Upon force application (10–25 pN), an unfolding event between LRR 2-4 was detected that enlarged the GPIbα concave binding pocket facilitating the interaction with VWF A1. This conformational change induced a prolonged bond lifetime compared to binding without an unfolding event [65]. How the LRRD and the juxtamembrane mechanosensitive domain (MSD) collaborate with each other to integrate the binding and pulling force into an intracellular biochemical signal triggering platelet activation was poorly understood. Recently, Ju et al. revealed that MSD is unfolded by increasing and constant forces whereas LRRD unfolding requires solely increasing forces [67]. Additionally, LRRD and MSD have different effects on Ca^2+^ release in platelets, whereas the former intensifies Ca^2+^ secretion and the latter determines the signal type (Fig. 3). The transduction of the mechanical domain unfolding into a biochemical signal affecting cytosolic Ca^2+^ release is at least in part mediated by protein 14-3-3ζ associated with GPIbα cytoplasmic tail. Blockade of the interaction between 14-3-3ζ and GPIbα reduced Ca^2+^ signaling.

Making the GPIbα mediated signal transduction even more complex, it is essential to consider the role of GPIbβ, since it is localized adjacent to the MSD in GPIbα. Different antibodies targeting the extracellular domain of GPIbβ can change the morphology of GPIbβ and have either potentiating or inhibitory effects on GPIbα signal transduction whereas ligand binding to GPIbα is not compromised [68–70]. GPIbβ deficient platelets reveal a decreased calcium signaling in response to a thromboxane analog or collagen-related peptide [71]. These results clearly indicate a modulatory role of GPIbβ for GPIbα signaling whereas the exact character of this interaction is still elusive. Taken together, advances in understanding the mechanosensitive domains in GPIbα could help to elucidate the mode of action of other mechanosensitive proteins or receptors in other cell entities in general.

### Intra-platelet signaling of GPIb-IX

When the vessel wall suffers damage, GPIb-IX is the first receptor that mediates an initial platelet adhesion to unfolded VWF even under high shear conditions typically found in the arterial microcirculation [72, 73]. Subsequent to adhesion, GPIb-IX initiates signaling cascades that trigger an inside-out activation of integrin α_IIb_β_3_ that in turn contributes to thrombus formation and stability [61]. Besides the enhancement of integrin affinity, GPIb-IX derived signals induce platelet degranulation, platelet shape change, desialylation, and microparticle and thromboxane A2 secretion [73–77].

The cytoplasmic tails of the GPIb-IX subunits have no enzymatic activities but interact with several intracellular proteins like filamin A, 14-3-3ζ, or calmodulin [25, 78, 79]. Filamin A anchors GPIbα to the cytoskeleton and maintains normal platelet integrity and shape and is vital for normal signal transduction reactions involved in platelet activation [79–81]. Several signaling molecules are activated by GPIb-IX upon ligand binding e.g., Src family kinase (Lyn, Fyn), Rac1 signaling pathway, phosphoinositide 3-kinase (PI3K)- Akt and cGMP-dependent protein kinase (PKG) pathway, mitogen-activated protein kinase (MAPK), and LIM kinase 1 pathway [82–85]. Interestingly, LIM 1 kinase seems to play a dual role in platelet activation, since LIM deficient platelets show an intensified response to PAR-4 agonist peptide or a TXA_2_ analog [85]. Furthermore, accumulating evidence suggests that the localization of GPIb-IX complex in glycosphingolipid-enriched membrane (GEM) domains is important for VWF mediated adhesion at high shear and platelet activation. Disruption of the disulfide-links between GPIbα and GPIbβ markedly decreased the amount of the GEM-associated GPIbα and impeded GPIbα mediated adhesion to VWF [86, 87]. Besides the localization in lipid raft domains, GPIb-IX exhibits a functional interplay with other platelet receptors e.g., GPVI, apolipoprotein E receptor 2, FcγRIIA receptor [88–90]. The co-localization and physical interaction with FcγRIIA receptor at the platelet membrane appears as an indispensable requirement for subsequent tyrosine phosphorylation and serotonin secretion due to VWF binding [90, 91]. Both receptors mutually cooperate and blocking one of the receptors affects signal transduction by the other [92, 93]. Upon VWF binding, GPIbα also associates physically with FcR γ-chain and forms a signalosome consisting of Src family kinase Fyn, and Lyn and tyrosine kinase Syk [94, 95]. The protein 14-3-3ζ is one of the best investigated intracellular ligands of GPIb, which has several bindings sites on GPIbα, also binds to the cytoplasmic tail of GPIbβ and is crucial for GPIb-IX mediated activation of integrin α_IIb_β_3_ [78, 96, 97]. Disruption of the interaction between GPIbα and 14-3-3ζ has an impact on thrombin-mediated platelet aggregation and granule secretion and also controls platelet apoptosis [59, 98]. In fact, enhanced association of GPIb with 14-3-3ζ proteins leads to release and activation of pro-apoptotic protein Bad from its binding to 14-3-3ζ and finally activation of caspase-9 [99]. Interestingly, the VWF binding function of GPIb-IX also depends on the cytoplasmic association of GPIb-IX with 14-3-3ζ [100, 101]. Considering these findings, one could argue that GPIb-IX has the ability to integrate intracellular signals to conformational changes in the outer membrane distal ligand-binding domain. This mechanism is thoroughly described for integrins and known as inside-out activation. Dai et al. proposed a “toggle switch model” as a possible regulatory mechanism for GPIb-IX in which 14-3-3ζ protein binds simultaneously to GPIbα and GPIbβ inducing a resting state of GPIb-IX. Conversely, dephosphorylation of GPIbβ results in binding of 14-3-3ζ to GPIbα alone and activation of the VWF binding function of the GPIb-IX complex [78, 100]. Nevertheless, this kind of affinity modulation of the GPIbα binding domain has to be regarded carefully since the signal has to be transmitted through a long and flexible MSD and sialomucin domain. Moreover, there are no reports available dealing with different states of affinity of the ligand-binding domain, making the inside-out activation of GPIb-IX in 14-3-3ζ toggle-like manner rather unlikely. Lastly, it is worth mentioning that protein 14-3-3ζ could reveal as an interesting pharmacological target since it is involved in GPIb-IX binding to VWF under high shear conditions and therefore has selective effects for arterial thrombosis.

### Relevance of GPIb-IX for platelet clearance

Platelets are the second most abundant cells in the blood circulation with a lifespan of 7–10 days in humans. Several billions of platelets are produced daily. To avoid spontaneous bleeding events or arterial or venous occlusions, the complex process of platelet production by megakaryocytes and clearance by hepatocytes, liver macrophages (Kupffer cells), or spleen macrophages is a sophisticated, balanced, and tightly regulated process [64]. During infection and sepsis, platelets are rapidly cleared from circulation to mitigate the risk of lethal coagulopathies [102]. The platelet lifespan is determined by anti-apoptotic Bcl-xL and proapoptotic molecules Bax and Bak, mitochondrial permeabilization, and phosphatidylserine exposure on the membrane which add to efficient platelet clearance.

Different pathways regulating hepatic platelet clearance have been identified in the last decades that also involve GPIb. First, patients with type 2D VWF disease usually suffer from thrombocytopenia and accelerated clearance of different severity since their VWF is mutated and has a constitutive affinity to GPIb. The complexes of mutant VWF and platelets are taken up by macrophages in the liver and spleen [103]. Also, the aminoglycoside ristocetin as well as the snake venom botrocetin increase the interaction between GPIb and VWF and can induce thrombocytopenia [104–106]. In patients with immune thrombocytopenia, autoantibodies against GPIb are responsible for enhanced platelet clearance considered as a reliable predictor for the success of therapeutic approaches [77, 107]. Two different models have been proposed to account for the aforementioned thrombocytopenic effects. First, the GPIb clustering model suggests a GPIb lateral dimerization in the platelet membrane due to VWF binding which offers multiple binding sites or by binding of one antibody to two GPIb receptors. As a consequence, the GPIb-IX complex is activated and transmits a signal through the platelet membrane [108, 109]. The second model, namely the trigger model, was proposed by Deng et al. in 2016 and confers the identification of the mechanosensitive domain to platelet clearance. In this model, the A1 domain of VWF binds to the ligand-binding domain of GPIb under physiological shear conditions and mediates the unfolding of the mechanosensitive domain and exposure of the juxtamembrane trigger sequence [14]. This newly identified trigger sequence consists of about 10 amino acids and is localized between the transmembrane domain and the mechanosensitive domain. Upon extension, the trigger sequence induces intracellular signaling and finally calcium influx, P-selectin, and β-galactose exposure on the platelet surface. Modifications of platelet glycans have a crucial impact on their clearance and lifespan. In particular, removal of terminal sialic acid from platelet glycans leads to exposure of multiple galactose residues on glycans which are subsequently recognized by the hepatic Ashwell-Morell receptor (AMR). The AMR is a multi-complex receptor and contains several lectin domains for interaction with galactose or galactosamine [110].

**Fig. 4:**
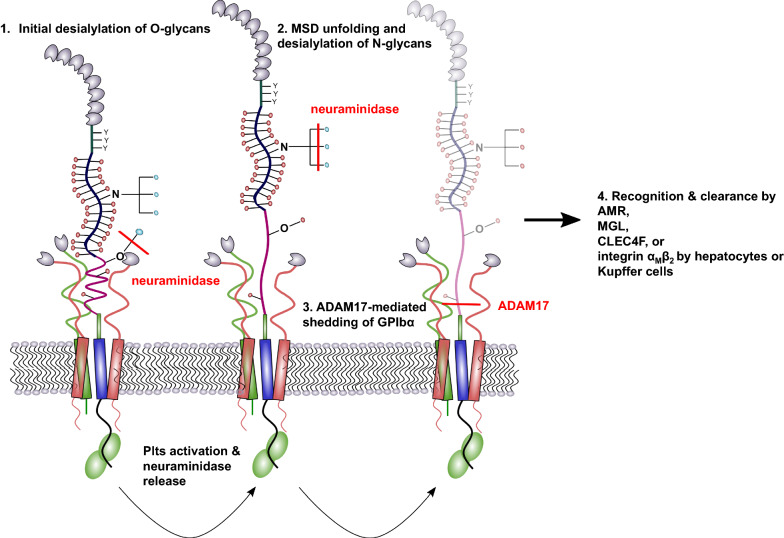
GPIbα in platelet clearance. Removal of terminal sialic acid from GPIbα *O*-glycans by neuraminidase leads to an unfolding event of the MSD and additional release of neuraminidases from platelet granules, which desialylate N-glycans on GPIbα. Finally, ADAM17 sheds desialylated GPIbα receptors from the platelet membrane. Platelets with desialylated residues expose galactose and N-acetylgalactosamine that is recognized by the AMR and leads to platelet clearance by hepatocytes and Kupffer cells

Binding to the AMR leads to platelet internalization and subsequent elimination [111]. Relatedly, platelet uptake by AMR stimulates hepatic thrombopoietin (TPO) generation and release which induces thrombopoiesis by megakaryocytes in the bone marrow to replace the eliminated platelets [112, 113]. It is a well-known fact that neuraminidases, enzymes that cleave the sialic acid from glycans, can induce transient thrombocytopenia. Treatment of platelets with neuraminidase ex vivo induced a rapid platelet clearance from circulation in rabbits and rats [114, 115]. In case of systemic infections with microbes expressing neuraminidases, platelets are progressively desialylated, expose penultimate galactose, and are recognized by the AMR [102]. Thus, it was shown that the AMR can alleviate sepsis-associated lethal coagulopathy. Furthermore, platelets themselves express neuraminidase in lysosomes, which is expressed on the platelet surface after activation and desialylates glycans culminating in platelet clearance from circulation and thrombocytopenia [77, 116]. GPIb is the second most abundant and the most heavily glycosylated platelet surface receptor with about 60% carbohydrate per weight [10, 77, 117, 118]. The carbohydrates are *N*- and *O*-linked and capped with sialic acid, which accounts for approx. 64% of the total platelet sialic acid [118]. Thus, desialylated GPIb was identified as the major target for AMR responsible for platelet clearance from the circulation [111]. Consequently, removal of the extracellular 45 kDa VWF binding domain of GPIb from long-term-refrigerated platelets restores their survival in the circulation. However, the elimination of the 45 kDa VWF binding domain did not completely rescue the platelet recovery in the circulation indicating that also other glycosylated platelet receptors, for instance integrin α_IIb_β_3_ are recognized and bound by the hepatic AMR [109]. The AMR exhibits a higher binding affinity and preference for tri- or tetra-antennary galactose-terminal oligosaccharides than for single galactose units [119–121]. Thus, it was supposed that galactose exposed by *N*-linked glycans is the major ligand for the AMR since *O*-linked glycans do not present a ternary complex [64]. Recently, Wang et al. revealed that desialylation of *O*-glycans, instead of *N*-glycans, on GPIbα induces mechanosensitive domain unfolding, GPIb-IX signaling, and finally platelet clearance by the AMR [122]. Subsequently, *N*-glycans are desialylated and amplify the platelet clearance (Fig. 4). These findings are supported by the notion that mouse GPIb does not contain any *N*-glycosylation sites but exhibit a similar drop in platelet count. Additionally, Li et al. revealed that mice lacking *O*-glycans exhibit reduced peripheral platelet numbers [123].

Hence, it is conceivable that mono-antennary *O*-glycans are the initial neuraminidase target expediting platelet clearance and that *N*-glycan desialylation occurs consecutively. Next to the AMR-mediated platelet clearance, also the integrin α_M_β_2_ and C-type lectin receptor, CLEC4F, on Kupffer cells have an impact on platelet phagocytosis as an alternative pathway for the elimination of senescent platelets from the circulation. The integrin α_M_β_2_ as well as CLEC4F recognize exposed and irreversibly clustered β-*N*-acetylglucosamine/*N*-acetylgalactosamine residues of *N*-linked glycans on GPIbα which initiates subsequent phagocytosis [20, 124, 125]. Furthermore, it is becoming increasingly clear that the Macrophage galactose lectin (MGL) supports AMR-mediated clearance by Kupffer cells [126].

Besides desialylation, also the cleavage of the whole ectodomain of GPIb is critically regarded to contribute to platelet clearance. This is especially relevant for platelets stored for subsequent transfusion. During storage, platelets undergo several structural modifications that alter their functional integrity. One of these changes is the metalloproteinase-mediated proteolysis and release of the GPIb ectodomain glycocalicin mentioned above, which is thus a signature event of platelet aging [127, 128]. GPIb shedding can be mediated by neutrophil-derived proteinase cathepsin G, the cobra venom mocarhagin or mainly by the metalloproteinase ADAM17 (also known as tumor necrosis factor-α converting enzyme) [106, 129, 130]. The specific cleavage site for ADAM17 lies in the mechanosensitive domain and leads to the exposure of the trigger sequence upon cleavage [14]. Thus, the detachment of the GPIb ectodomain by proteinases was associated with a reduction in platelet posttransfusion recovery and enhanced clearance from the circulation [128, 131, 132]. Metalloproteinase inhibition even prevented GPIb proteolysis on damaged platelets and improved the hemostatic functions of these cells in vivo [132]. However, Hoffmeister et al. showed that desialylation of GPIb precedes its cleavage by metalloproteinases and is thus responsible for platelet clearance after refrigeration. Furthermore, they elucidated that GPIb desialylation rather promotes ADAM17-mediated hydrolysis and receptor shedding even though that platelet clearance is mediated by desialylation and does not require GPIb shedding [133].

Concerning these findings, it is tempting to speculate that an ADAM17-mediated shedding of desialylated GPIb would rather prevent clearance since the exposure of galactose on the platelet membrane is reduced [109]. Therefore, further studies and in-depth experiments would help to elucidate the contribution and sequence of desialylation and shedding events in GPIb related platelet aging and clearance in the liver. Recently, Morodomi revealed another GPIb related platelet clearance pathway [134]. To study the mechanisms of immune thrombocytopenia, monoclonal antibodies targeting GPIbα were applied at different doses and routes of administration. A single, high-dose intravenous injection induced a platelet activation, aggregation, and finally desialylation. Desialylated platelets and aggregates got stuck in the microvasculature of the liver by binding to AMR and were phagocytosed as previously described in detail. Additionally, opsonized platelets are cleared by splenic macrophages via Fc receptor binding. In contrast, subcutaneous injection of low-dose antibodies every third day at three doses caused clearance of opsonized platelets primarily by splenic macrophages, whereas the liver remained almost completely unaffected. Obviously, the applied antibodies were unable to activate the platelets at a low-dose and to induce a neuraminidase expression which would culminate in desialylation and clearance in the liver. Thus, GPIbα is involved in the clearance of platelets in the spleen.

Although common mechanisms of platelet clearance in the liver and spleen have begun to emerge, several questions remain to be addressed in future studies for instance the counterreceptors for desialylated GPIb on hepatocytes and Kupffer cells.

### GPIbα and the hepatic thrombopoietin generation

Thrombopoietin (TPO) has been identified as a decisive factor for platelet production [135]. Decades later, the cognate receptor (c-Mpl, CD110) for TPO was found on megakaryocytes and a megakaryocyte proliferation and maturation due to TPO binding was demonstrated [136, 137]. TPO is not required for proplatelet production or shedding but it is critical for the maintenance of hematopoiesis and the hematopoietic stem cell niche [138, 139]. To balance the TPO levels in the circulation, it was a prevailing textbook concept for years that TPO is constitutively produced in the liver and regulated through binding and uptake by platelet and megakaryocyte expressed c-Mpl receptor [140, 141]. This theory was supported by the finding that a transfusion of non-manipulated platelets to mice resulted in an immediate and significant drop in TPO levels whereas the transfusion of TPO saturated platelets had no impact on TPO plasma levels [142]. Furthermore, in patients receiving chemotherapy, the serum TPO levels correlated inversely with platelet counts [143]. Grozovsky revealed an AMR mediated uptake of desialylated platelets which regulates the hepatic TPO mRNA expression in a JAK2-STAT3-dependent signaling pathway [112]. These findings are supported by previous observations dealing with an injection of neuraminidase treated and untreated platelets into rabbits. Desialylated platelets stimulated megakaryocytes to produce more platelets, whereas untreated platelets had no effect [144]. Based on this premise, Xu recently exhibited that the extracellular domain of GPIbα is responsible for platelet-mediated hepatic TPO generation [145]. GPIbα knockout platelets displayed an impaired binding to hepatocytes and failed to stimulate hepatic TPO production. Supportive of this finding, IL4R/GPIbα-tg mice (the extracellular domain of GPIbα is replaced with the IL-4 receptor α-subunit) had lower plasma TPO levels and IL4R/GPIbα-tg platelets failed to induce TPO generation in wild-type mice. Immobilization of recombinant GPIbα on beads stimulated hepatic TPO mRNA production similar to wild-type platelets and allows the conclusion that platelet GPIbα ectodomain alone, excluding other platelet factors or receptors, is sufficient to mediate hepatocyte TPO generation. In line with this, monoclonal antibodies targeting the N-terminus of GPIbα reduced the hepatic TPO production. A GPIbα desialylation on platelets further potentiated the TPO generation, however, it remained elusive whether the N-terminus alone can mediate the AMR response or whether parts or even the complete mucin-like region is required for TPO secretion. Finally, the authors speculate that also other AMR-independent GPIbα related pathways contribute to hepatic TPO production. However, the involved receptors on hepatocytes and ligands on GPIbα are still unclear.

### Role of GPIbα in platelet biogenesis

**Fig. 5:**
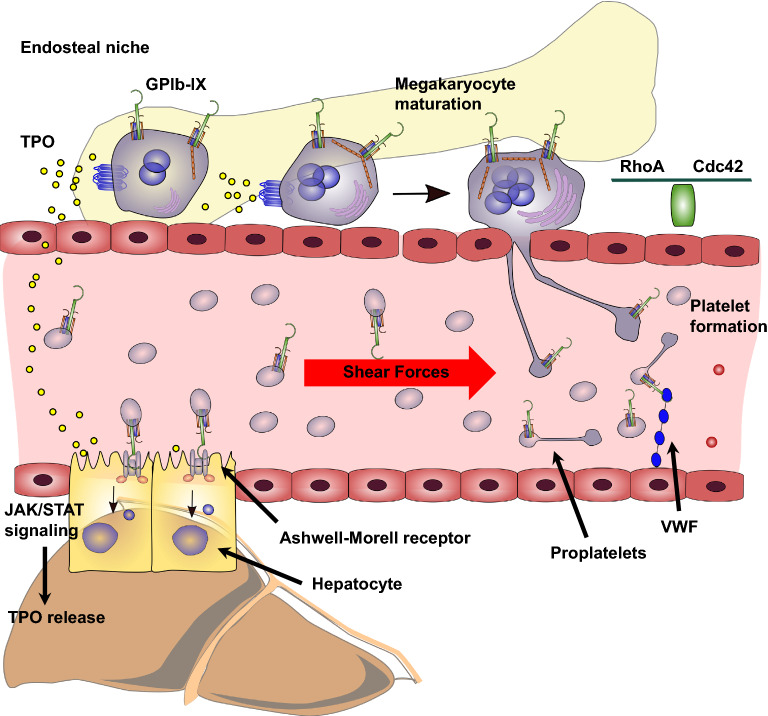
Role of GPIbα in platelet formation. GPIbα contributes to megakaryocytopoiesis and localization of megakaryocytes at the sinusoidal vessels. GPIbα regulates Cdc42 and RhoA activity, which is essential for a proper transendothelial proplatelet formation. Subsequently, proplatelets have to separate into platelets under high shear forces and this requires the binding of GPIbα to VWF. Aged and desialylated platelets bind to the hepatic Ashwell-Morell receptor (AMR) which leads to JAK2/STAT3 signaling and finally thrombopoietin generation and secretion from hepatocytes. Thrombopoietin, in turn, expedites megakaryocyte maturation and proplatelet formation

Megakaryocytes are the largest cells in the bone marrow (50–100 µm) and responsible for the production and secretion of about 10^11^ platelets per day [64, 146]. Megakaryocytes develop from hematopoietic stem cells under the continuous supply with TPO and further regulatory proteins [136, 138, 147, 148]. After normal proliferative events megakaryocytes start with endomitosis and accumulate DNA content ranging from 4 n to even 128 n with a median content of 16 n [149]. The endomitotic process is at least partially mediated by a downregulation of the small GTPase RhoA, which normally regulates the F-actin formation in the cleavage furrow [150]. The result of the endomitosis is polyploidy that is in turn required for the generation of the invaginated membrane system (also known as demarcation membrane system) that deals as a reservoir for proteins, lipids, and membrane components [151, 152]. Subsequently, mature polyploid megakaryocytes localize from the endosteal niche towards sinusoidal bone marrow endothelial cells triggered by stromal cell-derived factor-1 and CXCR4 [153–155]. There they undergo complete cytoskeleton reorganization with microtubule and actin involvement, to induce long pseudopodial elongations called proplatelets, the precursors of platelets [156]. Once proplatelets have reached the bloodstream in the vascular sinusoidal circulation, they are released heterogeneous in size, convert reversibly to preplatelets, and finally mature to platelets after a final abscission event [157, 158]. Several steps in the maturation cascade form hematopoietic stem cells to circulating platelets are under the control or at least affected by GPIb. In the first studies investigating this issue, megakaryocytes from Bernard–Soulier syndrome patients or GPIb deficient mice revealed a poorly developed demarcation membrane system and other ultrastructural changes compared to wild-type megakaryocytes [159, 160]. In another in vivo study, GPIbα deficiency resulted in a defect megakaryocytopoiesis associated with a disordered platelet developing field. GPIbα-deficient mice exhibited normal hematological profile except for platelet number and size [161]. Generation of IL4R/GPIbα-tg mice with an extracellular IL4 receptor and an intracellular GPIbα cytoplasmic tail revealed a two-fold increase in circulating platelet count and a 50% reduction in platelet size when compared with platelets from the GPIbα-deficient mouse model [162]. These data provided the first evidence that the GPIbα cytoplasmic tail is crucially involved in megakaryocytopoiesis and platelet generation [163]. Nevertheless, also the ectodomain of GPIbα seems to have an impact on megakaryocytopoiesis since antibodies directed against GPIbα inhibited megakaryocyte colony formation as well as proplatelet formation [164, 165]. To further focus on the impact of the cytoplasmic tail of GPIb for plateletpoiesis, the association with the membrane skeleton and scaffolding protein filamin A was explored. The interaction of filamin A with the GPIb complex is important for some cellular functions like GPIb biosynthesis, processing, and adhesion under flow conditions [166–168], whereas the role in platelet generation remained elusive. Kanaji recently revealed that a distinct ratio of GPIb and filamin A expression is required for an efficient transit of either protein to the cellular membrane and subsequent formation of platelets from proplatelets [169]. It is supposed that GPIb directs filamin A to the inner face of the membrane where it can become incorporated into the membrane skeleton and regulate the deformability of the proplatelet membrane during thrombogenesis. In the absence of filamin or GPIbα, megakaryocytes may not possess sufficient contractile force to restrict proplatelet size, resulting in an aberrant, early release of giant platelets from their proplatelet precursors [169]. To further explore the contribution of the GPIbα cytoplasmic tail to megakaryocytopoiesis and platelet secretion, Ware et al. generated transgenic mice lacking the 6 terminal residues (605–610) on the cytoplasmic tail of human GPIbα critical for binding to the signal transduction protein 14-3-3ζ. In a model of severe thrombocytopenia or after stimulation with TPO, they found that a high percentage of megakaryocytes with truncated GPIbα cytoplasmic tail had amplified polyploidy (32 n versus 16 n for wild-type megakaryocytes) and were more differentiated compared to wild-type megakaryocytes. In contrast, the latter have a greater proliferative potential [170]. Focusing on the underlying molecular mechanism, an increased PI3K/Akt phosphorylation was detectable in megakaryocytes in which the interaction between GPIbα and protein 14-3-3ζ was impeded. Finally, the authors propose the hypothesis that the interaction between GPIbα cytoplasmic tail and protein 14-3-3ζ down-regulates Akt activity thereby controlling endomitosis and ploidy in megakaryocytes. This is supported by previous findings from Bialkowska et al. exhibiting that when present in cultured CHO cells, the cytoplasmic domain of GPIbα can sequester sufficient endogenous 14-3-3ζ to almost totally inhibit integrin-induced Cdc42 and Rac activation and finally cell spreading [171]. Recently, Nieswandt et al. revealed in an elegant story that the megakaryocyte localization at sinusoids and polarization of protrusions to secrete proplatelets is regulated by small GTPases RhoA and Cdc42, both limiting each other [172]. The activity of Cdc42 is controlled by GPIb and PI3K signaling and a blockade results in a reduced megakaryocyte localization at the sinusoids and an increased population in the bone marrow hematopoietic compartment. Conversely, RhoA-deficiency leads to megakaryocyte hyperpolarization and complete transmigration through the endothelial barrier into sinusoidal vessels (Fig. 5). Thus, GPIb signaling controls the Cdc42/RhoA regulatory circuit and consequently regulates the transendothelial platelet biogenesis.

At the almost final step of platelet generation, proplatelets have to separate from the megakaryocyte cell body as mentioned before. Accumulating evidence from several reports suggests that this process is strongly facilitated by shear forces similar to those in the bone marrow sinusoids [173]. Dunois-Lardé et al. illustrated that an initial adhesion of megakaryocytes to VWF under high shear forces (1800s^−1^) is mediated by GPIb. The subsequent formation of platelets from proplatelets was completely abrogated by antibodies targeting the VWF-GPIb interaction whereas integrin a_IIb_b_3_ is not required for proplatelet generation [174]. The applied shear rate of 1800s^−1^ resembles those in small arterioles and capillaries of the pulmonary circulation and is higher than the ones existing in murine bone marrow sinusoids (50–165 s^−1^) [175, 176]. The authors suggest that the lung circulation could be an important site of platelet production, as the lung capillaries are the first to be encountered by cells from the bone marrow [177]. Furthermore, these results emphasize the contribution of the extracellular domain of GPIb for platelet formation and support the findings of Kanaji et al. exhibiting a mild form of thrombocytopenia in transgenic mice with an extracellular IL4 receptor connected to the cytoplasmic domain of GPIb as mentioned previously [162]. These results are also in line with Balduini et al. who showed proplatelet formation exclusively prevented by blockade of GPIb-IX-V, or upon cleavage of GPIba by the metalloproteinase mocarhagin [178]. Thus, it is conceivable that thrombocytopenia in Bernard–Soulier syndrome patients is also caused by incompletely fragmented proplatelets due to reduced GPIb adhesion capacities under shear forces although the number of the bone megakaryocytes is normal. The importance of a fine-tuned interaction of GPIb with VWF for a proper platelet formation is further corroborated by findings from patients with type 2B VWF disease. These patients have a VWF gain-of-function mutation and therefore an enhanced binding of GPIb to VWF associated with a transient macrothrombocytopenia [179]. Thus it is tempting to speculate that the tight binding causes a slower and less efficient process of platelet formation at high shear rates, responsible for an increased proportion of giant platelets detected in these patients [174, 180, 181].

In summary, GPIb is obviously involved in different steps of thrombopoiesis ranging from fine-tuning of TPO signaling during megakaryocyte maturation to the generation of contractile forces in conjunction with filamin A and finally regulation of Cdc42/RhoA activity during transendothelial platelet biogenesis. The GPIb extracellular domain seems especially important for shear-mediated proplatelet separation, whereas the cytoplasmic tail is integrated into signaling and forms a stable contact to the platelet cytoskeleton.

Thus, it is plausible that further, up to now unknown GPIb-IX associated mechanisms are existing with relevance for the process of platelet generation and which potentially reveal therapeutic options for patients with thrombocytopenia.

### Immunological functions of GPIb-IX

It is becoming increasingly clear that platelets are also involved in a plethora of immunological processes extending beyond the paradigm of platelets as simple mediators of aggregation and clot formation. Platelets express a range of functional immune receptors on their surface and contain an abundance of bioactive proteins in their α- and dense granules. Thus, platelets have numerous possibilities to communicate with and impact other cells effectively in their particular functions. For instance, they are involved in the antimicrobial host defense, allergic inflammation, in the modulation of the innate and adaptive immune response, and the process of antigen presentation in many different ways. The current understanding of the immunological functions of platelets has been summarized in several excellent reviews recently [182–186]. Notably, GPIb is a key player that participates in, or solely mediates these immunological functions of platelets.

Neutrophil extracellular traps (NETs) are webs of histone-modified nuclear material and proteins containing elastase and myeloperoxidase secreted from activated neutrophils during an inflammatory response [187]. NETs bind, ensnare, and kill Gram-positive and -negative bacteria and degrade virulence factors. Moreover, NETs were found in several non-infectious inflammatory diseases like thrombosis, small-vessel vasculitis, or myocardial infarction among many others [188–191]. To induce a NET release from neutrophils, platelets have to be activated either with lipopolysaccharides (LPS) binding to their Toll-like receptors 4 (TLR4), by TLR1/2 agonists, or by the classical agonists such as thrombin or collagen [192]. Upon activation, several platelet-derived pro-inflammatory mediators like high-mobility group box 1 (HMGB1), chemokine CXCL4, or β-defensin have been identified to trigger NETosis from neutrophils [190, 191, 193]. Furthermore, a direct interaction between platelets and neutrophils seems to be a prerequisite for NET secretion. Platelet expressed P-selectin and β_2_-integrin have been identified to mediate direct contact with neutrophils, nevertheless further molecular pathways are poorly defined. Recently, von Brühl et al. revealed that the GPIbα-dependent interaction of platelets with neutrophils exerts several functions during deep vein thrombosis development. First, GPIbα is responsible for the overall platelet recruitment during initial thrombus formation. Second, GPIbα supports leukocyte recruitment and ultimately, it instigates NET formation from neutrophils [194]. These results were corroborated by Carestia et al. who found a reduced NET release upon GPIb inhibition on platelets or β_2_-integrin on neutrophils [195, 196]. Furthermore, a blockade of VWF markedly reduced platelet-mediated NET formation. Thus, it is conceivable that after secretion from platelet granules, VWF may act as a bridge bringing platelets and neutrophils into proximity[197]. Another highly relevant mechanism of how platelets and GPIb support the innate immune surveillance to clear Bacillus cereus and Staphylococcus aureus from the blood circulation was revealed by Kubes et al. [198]. Applying multichannel intravital spinning-disk confocal microscopy, they identified a previously unknown patrolling mechanism of platelets in the blood that involved permanent ‘touch-and-go’ interactions of GPIb with constitutively expressed VWF on Kupffer cells in liver sinusoids under basal conditions. After Bacillus cereus injection, the constitutive VWF expression in the liver vasculature significantly increased once microbes were caught by Kupffer cells. Subsequently, the transient GPIb-mediated interactions between Kupffer cells and platelets were converted to profound sustained binding of platelets to the Kupffer cell surface with subsequent aggregation and encasement of the bacteria by the platelets. The firm adhesion of platelets to the Kupffer cells was mediated by integrin a_IIb_b_3_ and Kupffer cell-expressed VWF. In fact, more than 80% of GPIb deficient mice died within the first 4 h of infection with Bacillus cereus. In contrast, there was less than 10% mortality for wild-type counterparts. Thus, these findings show that the platelet GPIb-mediated, permanent surveillance of Kupffer cells in the liver is responsible for a quick and efficient killing of ingested microbes long before classical effector cells of the innate immune response are recruited.

For the clearance of the Gram-positive, facultative intracellular bacterium Listeria monocytogenes from the blood circulation, a platelet GPIb, complement C3, and CD8^+^ dendritic cell (DC)-dependent pathway has been identified and supports the notion that platelets also participate in the formation of the adaptive immune response [199]. In detail, bacteria were opsonized with complement factor C3 upon their entry in the circulation. Afterward, platelets bind via GPIb to these bacteria targeting them to the spleen where they were taken up by splenic CD8α^+^ DCs finally generating an adaptive T cell mediated response. GPIb and platelets are part of an efficient shuttling system for blood-borne bacteria to a potent immunity-inducing DC population that simultaneously provides an early survival niche. As expected, platelet depletion led to the impaired shuttling of Listeria monocytogenes to CD8α^+^ DCs but induced an initial accelerated clearance from the bloodstream. Thus, platelets and GPIb regulate rapid destruction of systemic bacteria by phagocytes in the spleen, while diverting a small but important fraction of viable bacteria to the immunity-inducing CD8α^+^ DC compartment.

GPIbα is also involved in the trafficking of leukocytes to the site of thromboinflammation. Upon vascular injury in the mesenteric venous circulation of mice, α-thrombin is generated which induces platelet activation, thrombus formation, and finally leukocyte recruitment and invasion through the body of the thrombus. In this cascade, the activity of α-thrombin is regulated by GPIbα on platelets. If α-thrombin is unable to bind to a mutated GPIbα ectodomain, the thrombus size is increased and also the number of leukocytes invading the thrombus is enhanced. Thus, the α-thrombin-binding function of both GPIbα serves to limit the availability of catalytically active thrombin and prevents excessive thrombus formation and leukocyte recruitment to sites of vascular injury [200].

Next to its function as a regulator of thrombin activity, also the adhesive function of GPIbα contributes to the recruitment of inflammatory monocytes to sites of endothelial perturbation in large and small blood vessels [201]. Upon platelet activation by different stimuli, platelet-derived extracellular vesicles are shed and mounting evidence supports a role of these particles in different inflammatory diseases like type 2 diabetes, acute coronary syndrome, or atherosclerosis among others [202–204]. One mechanism of how these microparticles contribute to inflammatory processes was recently revealed by Rainger et al.. The microparticles bind preferentially to classical monocytes in a P-selectin and phosphatidylserine-dependent fashion and progressively transfer GPIbα to the monocyte membranes. GPIbα from platelet-derived microparticles supports monocyte rolling on VWF, recruitment, and adhesion on TGF-β1 stimulated endothelial cells, and ultimately recruitment to the TGF-β1 stimulated vasculature in the cremaster muscle in vivo. Surprisingly, neutrophils and lymphocytes do not incorporate GPIbα containing particles in their membranes. This data adds another important aspect in understanding the contribution of platelet GPIb to thromboinflammatory pathology. The tight relation between platelets and monocytes is also reflected in a recent study from Schattner et al. showing that platelets can drive the polarization of monocytes in presence of LPS towards an M1 inflammatory phenotype, whereas LPS alone polarizes monocytes towards anti-inflammatory M2 macrophages [205]. An initial and direct binding event between platelet GPIb and monocyte/macrophage integrin subunit CD11b in addition to platelet released PF4 is responsible for the monocyte-macrophage differentiation and polarization reprogramming. Validating these results in vivo in two different sepsis models, a platelet transfusion up to 6 h after induction of infection reduced mortality and increased the percentages of iNOS^+ ^macrophages. Blocking either CD11b or GPIb significantly reduced the survival rate of septic animals and iNOS^+ ^cells in the spleen.

Besides shaping the immune response for an efficient bacterial clearance, GPIbα and its counterreceptor leukocyte integrin Mac-1 are also involved in the pathogenesis of experimental autoimmune encephalomyelitis which resembles Multiple Sclerosis in humans. This autoimmune disease is characterized by the infiltration of the neuronal tissue by T-cells autoreactive to antigens of the myelin sheath, subsequent breakdown of the blood–brain barrier, and the recruitment of further inflammatory effector cells such as mononuclear cells and macrophages. Finally, the activation of resident inflammatory microglial cells contributes to the development of CNS lesions [206–208]. Platelets are present in Multiple Sclerosis lesions and promote leukocyte recruitment to the inflamed spinal cord, and the upregulation of multiple inflammatory cytokines, chemokines, and adhesion molecules [209]. Treatment of mice with a Fab to GPIbα after disease onset resulted in a transient but significant clinical recovery from encephalomyelitis as compared with Fab control experiments. Blockade with an antibody that was raised against the binding site of Mac-1 for GPIbα without affecting other Mac-1 adhesive interactions resulted in a prolonged reduction of clinical encephalomyelitis symptoms as compared with the control antibody administration. Thus, GPIbα is crucially involved in the progression of an autoimmune disease at least in a mouse model of encephalomyelitis.

**Fig. 6:**
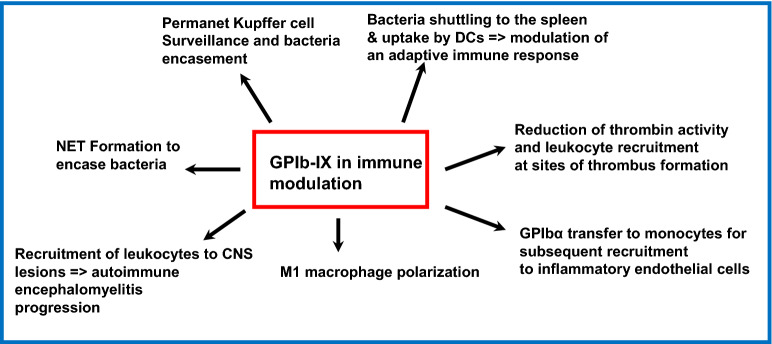
Multiple roles of GPIb-IX in immune modulation. GPIb-IX regulates NET formation, bacteria shuttling to the Kupffer cells and uptake by dendritic cells, and is involved in bacteria encasement. GPIb-IX regulates thrombin activity at sites of immune thrombosis, initiates macrophage polarization towards an M1 phenotype, and supports monocyte recruitment to inflammatory endothelial cells. GPIb-IX regulates leukocyte recruitment to the central nervous system which contribute to severity of autoimmune encephalomyelitis

A considerable amount of work has shown the impact of platelets on the shape of the innate as well as the adaptive immune response. Notably, GPIb seems to be a key receptor in this context, since it mediates bacteria shuttling, NETosis, adhesive functions of monocytes, macrophage polarization, and finally regulates thrombin activity. The different immune related mechanisms that are modulated by GPIb-IX are summarized in Fig. 6. Thus, it is likely that in future studies, novel mechanisms will be detected that will help to fully comprehend how GPIb-IX impacts the immune response under physiological and pathophysiological conditions.

### GPIb in cancer metastasis and liver cancer development

The contribution of platelets to cancer progression and metastasis has long been recognized and is clinically reflected by the fact that cancer patients have an increased risk to suffer from venous thromboembolism compared to healthy individuals due to a hypercoagulable state [210, 211]. A multitude of studies has provided evidence of a mechanistic link between tumor cell dissemination and platelet activation. In fact, the ability of tumor cells to induce platelet aggregation was correlated with the metastatic potential of the tumor cells in in vivo experiments [212, 213]. During the last decades, a lot of different mechanisms on how platelets promote cancer metastasis have been identified. In brief, after tumor cell invasion in the blood circulation, platelets are immediately activated and encase the tumor cells to protect them from shear forces and the clearance by NK cells [214–216]. Platelet-derived mediators and the direct contact to the tumor cells can change the tumor cell phenotype from an epithelial to a pro-metastatic mesenchymal one [217]. Platelet secreted chemokines recruit granulocytes to the tumor cells which in turn foster the development of an early metastatic niche [218]. Platelets mediate the tumor cell adhesion to the vascular wall and facilitate the subsequent extravasation into subendothelial tissues [219–221]. Ultimately, platelet-derived growth factors expedite cancer cell proliferation, angiogenesis, and prevent intratumoral hemorrhage [222–224]. The contribution of GPIb-IX to the different steps of the metastatic cascade and cancer progression was the subject of various studies in the past decades with controversial outcomes. In early in vitro experiments, GPIbα was attributed to induce a platelet aggregation upon tumor cell contact [225, 226], while other groups did not support this assumption [212, 227].

Interestingly, although GPIbα was originally thought to be exclusively expressed on platelets and the megakaryocytic lineage, the aberrant expression of GPIb on different cell entities for instance breast cancer cells was reported and functionally investigated [228–231]. Breast cancer expressed GPIb together with VWF contributed to tumor cell aggregation, tumor cell spreading and filopodia formation on VWF, and finally enhanced cancer cell transmigration. Nevertheless, cancer cell expressed GPIb is rather uncommon and should be regarded as a cell line-specific exception.

Furthermore, the assistance of platelet GPIbα to metastasis remained elusive, since in vivo studies also obtained controversial results. Ware et al. observed reduced pulmonary metastasis upon melanoma cell tail vein injection in GPIb-deficient mice or IL4R/GPIbα-tg mice. However, direct binding between melanoma cells and platelets was not different between GPIb wild-type, GPIb-deficient, or IL4R/GPIbα-tg platelets [232]. Supportive of these findings, Qi et al. applied GPIb directed antibodies and found a reduced number of surface nodules in experimental and spontaneous metastasis models applying Lewis lung carcinoma, melanoma, or breast cancer cells [233]. Notably, the antibodies markedly reduced the interaction between cancer cells, platelets, and endothelial cells in in vitro experiments. In striking contrast, Erpenbeck reported a significant increase in pulmonary metastasis of melanoma cells after GPIbα inhibition with monovalent Fab fragments [234]. Especially, blocking of GPIbα before, or simultaneous with tumor cell injection seemed to comprise a survival advantage for the melanoma cells.

Interestingly, GPIbα blockade in P-selectin-deficient mice had no enhancing effect on metastasis. Since P-selectin is known for its ability to mediate the interaction with the endothelium [235], it is conceivable that a blockade of platelet GPIbα enhances the accessibility of P-selectin to adhere to the lung vasculature and establish a metastatic nodule. Thus, the role of GPIb in hematogenous metastasis is hitherto unclear and obviously differs with the applied experimental settings and warrants further investigations.

For tumor growth, the formation of new and stable blood vessels is a prerequisite to supply the tumor tissue with oxygen and nutrition. Besides platelet´s function to induce angiogenic vessel formation by secretion of pro-angiogenic factors, also platelet adhesion to angiogenic vessels mediated by GPIbα supports angiogenesis and prevents excessive leakage and hemorrhage from newly formed vessels [222]. Thus, GPIbα seems to be involved in platelet-mediated angiogenesis in two different fashions. Of note, GPIbα on extravasated platelets, localized in the tumor tissue, has also been utilized as a maker for epithelial-mesenchymal transition and tumor progression [236]. GPIbα was primarily found on the invasive front of the tumor and its expression correlated with the epithelial-mesenchymal transition transcription factor Snail 1.

Finally, in a seminal work, Heikenwälder recently revealed that platelet GPIbα crucially contributes to the transition from non-alcoholic fatty liver to steatohepatitis to liver cirrhosis and finally to hepatocellular carcinoma development in mice [237]. Platelets are well-known as active players in liver disease and inflammation and it has been reported that activated platelets contribute to cytotoxic T lymphocyte-mediated liver damage [238–240].

In the mentioned study, they found an early accumulation of activated platelets in the liver due to a high-fat diet accompanied by immune cell infiltration and activation of potential inflammatory pathways supporting hepatocarcinogenesis. Platelet inhibition with aspirin and clopidogrel abrogated immune cell infiltration into the liver and prevented hepatocellular carcinoma development. The intrahepatic interaction of platelets with Kupffer cells was mediated by GPIbα, and a short GPIbα blockade significantly reduced intrahepatic platelet accumulation. Consequently, steatosis, liver damage, and intrahepatic immune cell infiltration were attenuated and fibrosis was dampened. Similarly, IL4R/GPIbα-tg mice fed with a high-fat diet revealed a significant reduction in intrahepatic CD8^+^ T cell and NKT cell infiltration and lacked any macro- or microscopic evidence of liver cancer after 12 months. In particular, blockade of known GPIbα ligands P-selectin, VWF, or Mac-1 had failed to block steatohepatitis development. Notably, blocking platelet-derived GPIbα is a possible therapeutic approach to revert non-alcoholic steatohepatitis, but also to prevent the transition to liver cancer.

Thus, it is becoming increasingly clear that GPIb-IX is involved in many key steps of cancer progression, ranging from prevention of hemorrhage to development of hepatocellular carcinoma although the exact role in hematogenous metastasis is still ambiguous.

## Conclusion

In the last years, eminent progress has been made in the understanding of the physiological function of the GPIb-IX complex. It became evident that GPIb-IX is involved in platelet clearance, platelet formation, thrombopoietin generation and thus is a key molecule controlling the platelet equilibrium. Furthermore, GPIb-IX deals as a real thrombin receptor in platelet aggregation, is involved in several immunological mechanisms, and crucially contributes to liver cancer development. Despite this gain of knowledge, further in-depth characterization of GPIb-IX could reveal additional physiological and pathophysiological mechanisms that are at least partially regulated by GPIb-IX. Owing to GPIb-IX´s role in the first interaction between platelets and damaged vessels at sites of arterial stenosis where blood flows with high shear rates, GPIb-IX is an attractive target in attenuating thrombosis. Several inhibitors are currently tested as novel anti-platelet approaches with the assumption that they will have a favorable profile compared to state-of-the-art drugs like aspirin, clopidogrel, and tirofiban [241–243].

Nevertheless, with an increasing insight in the multiple GPIb-IX related mechanisms, it is conceivable that severe side effects could occur upon GPIb blockade. In contrast, GPIb-IX could reveal as interesting and relevant target for the treatment of other diseases than arterial and microvascular thrombosis.

## Data Availability

The material supporting the conclusion of this review has been included within the article.

## Abbreviations

AMR: Ashwell-Morell receptor
DC: Dendritic cell
GEM: Glycosphingolipid-enriched membrane
LBD: Ligand binding domain
LRRD: Leucine-rich repeat domain
MAPK: Mitogen-activated protein kinase
MGL: Macrophage galactose lectin
MSD: Mechanosensitive domain
NET: Neutrophil extracellular traps
PAR: Protease-activated receptor
PI3K: Phosphoinositide 3-kinase
TPO: Thrombopoietin
VWF: von Willebrand factor
